# Functional goals and outcomes of rehabilitation within palliative care: a multicentre prospective cohort study

**DOI:** 10.1186/s12904-025-01816-0

**Published:** 2025-07-01

**Authors:** Matthew Maddocks, Lucy Fettes, Naomi Takemura, Joanne Bayly, Helena Talbot-Rice, Karen Turner, Rebecca Tiberini, Richard Harding, Fliss E.M. Murtagh, Richard J. Siegert, Irene J. Higginson, Stephen A. Ashford, Lynne Turner-Stokes

**Affiliations:** 1 Cicely Saunders Institute of Palliative Care, Policy & Rehabilitation, Faculty of Nursing, Midwifery & Palliative Care, King’s College London, London, UK; 2 School of Nursing Li Ka Shing Faculty of Medicine, The University of Hong Kong, Pokfulam, Hong Kong China; 3 St Christopher’s Hospice, 51-59 Lawrie Park Road, Sydenham, London, UK; 4 Royal Free London NHS Foundation Trust, Pond Street, London, UK; 5 Rebecca Tiberini Consultancy and Coaching, St Moritz, Switzerland; 6 Wolfson Palliative Care Research Centre, Hull York Medical School, University of Hull, Hull, UK; 7 Department of Psychology and Neuroscience, Faculty of Health and Environmental Science, Auckland University of Technology, Auckland, New Zealand; 8 Regional Hyper-Acute Rehabilitation Unit, Northwick Park Hospital, London North West University Healthcare NHS Trust, London, UK

**Keywords:** Rehabilitation, Functioning, Goals, Palliative care, End of life

## Abstract

**Background:**

Rehabilitation is an integral component of palliative care. An understanding of functional goals can help tailor interventions and support the evaluation of services. This study examined the nature and timescale of functional goals in palliative care, attainment of goals following personalised rehabilitation, responsiveness relative to health-related quality of life across, and factors associated with goal achievement.

**Methods:**

Prospective, observational cohort study of adults with advanced progressive illness from 10 UK hospices referred for rehabilitation assessment. Urgency of care needs and functional status were assessed using the palliative Phase of Illness (stable, unstable, deteriorating) and Australia-modified Karnofsky Performance Status (AKPS, ≥ 60,60 − 50, ≤ 40) respectively. Health-related quality of life was assessed using EuroQoL 5-Dimension 5-Level (EQ-5D-5 L) utility score. Functional goals were set collaboratively with patients using SMART goal statements, mapped onto the WHO International Classification of Functioning, Disability and Health (ICF). Goal Attainment Scaling (GAS) was used to evaluate achievement against an anticipated outcome using a T-score. Ordinal logistic regression was sued to identify factors associated with goal achievement.

**Results:**

364 patients (54% female, mean (SD) age 68 (14) years, 71% cancer, 71% stable Phase, median AKPS 60) took part. They set a median (range) of 2 (1–4) goals; 645 in total. Goals had a median (range) timeframe of 28 (1-196) days and spanned 13/30 ICF domains; most frequently mobility, general tasks and demands, mental functions, community, social and civic life, and self-care. The majority focused on activity (51%) and participation (20%). Following personalised rehabilitation, GAS T-scores improved overall (mean (SD) change 8.9 (13.4)) and for each subgroup by Phase and AKPS (all *p* < 0.01). EQ-5D scores improved overall, but not for those with a deteriorating Phase or AKPS ≤ 40. Living alone or receiving multiple interventions increased the likelihood of goal achievement, whereas being wheelchair or bedbound, receiving a generic exercise intervention, or having goals rated as very difficult reduced it.

**Conclusions:**

Functional goals in palliative care typically focus on optimising activity and participation in the short term. Progress towards personalised goals can be achieved through personalised rehabilitation, including among people with deteriorating health or largely confined to bed. Goal Attainment Scaling can help direct and evaluate rehabilitation interventions in this setting.

**Supplementary Information:**

The online version contains supplementary material available at 10.1186/s12904-025-01816-0.

## Background

Over 73.5 million worldwide experience suffering related to serious life-threatening or life-limiting illness [1]. Physical symptoms of pain, breathlessness, fatigue and weakness represent two-thirds of this suffering [2], and the loss of function that affects this population contributes towards the 2.41 billion people globally that would benefit from rehabilitation [3].

Palliative care aims to enhance the quality of life of people affected by serious illness through person-centred care aligned to each individual’s concerns, problems and priorities [4]. Most people living with serious illness want to continue in usual roles and routines, stay mobile, socially active, and avoid depending on others for daily activities and self-care [5–7]. To this end, the provision of rehabilitation– processes of care to optimise functioning - is increasingly recognized as an integral component of palliative care [8, 9]. Rehabilitation in palliative care aims to empower people to self-manage their condition, reduce the impact of symptoms and optimise independence [8, 10]. Rehabilitation interventions in this setting are varied but include education and advice, symptom management techniques, assistive technology, occupational adaption, and planning for the future [11, 12].

Understanding functional goals is an essential to the rehabilitation process and person-centred care [13]. This involves an iterative process of identifying aspects of function important to the person, setting and negotiating goals, then planning rehabilitation activities and evaluations [14]. Active patient involvement in goal setting improves motivation and engagement in rehabilitation activities and can improve outcomes [15, 16]. Goal setting in the rehabilitation process can also lead to higher levels of self-efficacy and quality of life [15]. By capturing the aspects of function important to each patient, the rehabilitation process can be tailored in response to their priorities [17, 18]. There is limited evidence around functional goals in palliative care [19–22]. This may reflect the complex of care which balances the dual priorities of living well and preparing for end of life [20, 23]. Consequently, the use of goal setting to understand, direct and evaluate rehabilitation in palliative care is poorly articulated [24].

Therefore, this study aimed to: (i) determine the nature and timescale of functional goals in palliative care; (ii) assess goal attainment achieved through personalised rehabilitation; (iii) evaluate the responsiveness of goal attainment scaling relative to health-related quality of life, across palliative Phase of Illness and functional status; and (iv) explore patient, service, and goal–related factors that associated with goal achievement.

## Method

### Study design and settings

For this prospective, observational cohort study, a consecutive series of inpatients and outpatients were approached and recruited in 10 hospices throughout the United Kingdom. Sites offered rehabilitation interventions as part of usual care across inpatient, outpatient or community services. No pre-specified rehabilitation service or intervention was required for a site to participate in recruitment. Rehabilitation was recorded by setting (inpatient, outpatient, community), providers (physiotherapists (PT), occupational therapists (OT), speech and language therapists (SALT), dietitian) and intervention type (equipment provision and training; task practice; exercise; mobility practice; socialisation and communication; symptom management; other). Intervention categories were developed collaboratively with site staff and patient and public members.

### Study population and recruitment

Eligible patients were: (i) adults aged 18 years or above; (ii) diagnosed with an advanced or progressive illness; (iii) referred for rehabilitation assessment due to functional limitation; and iv) able to provide written informed consent in English language. Consecutive eligible patients were provided with a Participant Information Sheet by a member of the direct clinical team, then given opportunity to ask any questions and consider taking part. Interested patients provided written informed consent before study commencement in compliance with the Declaration of Helsinki. Study procedures were approved by the Cambridge East Research Ethics Committee (REC Reference: 16/EE/0031).

### Measurements and procedures

#### Sociodemographic and clinical characteristics

Patients’ age, sex, primary diagnosis and co-morbidities, and living arrangement (alone or with caregiver), were documented at baseline. Functional status was evaluated using the Australia-modified Karnofsky Performance Status (AKPS) [25], an 11-point scale ranging from 0 (deceased) to 100 (best possible function). Urgency of palliative care needs were characterised by the ‘palliative Phase of Illness’ classification, as determined by clinicians [26]. The phases reflect urgency of palliative care needs, and are defined as ‘stable’, ‘unstable’, ‘deteriorating’, ‘dying’ or ‘deceased’.

#### Goal setting and evaluation

Goal Attainment Scaling (GAS) was used to set goals and to assess the extent to which the goals set were achieved. GAS was introduced by Kiresuck and Sherman in the 1960s [27] and has been widely applied in rehabilitation [28, 29]. Utilising the GAS ‘light’ method [29], the multidisciplinary team and patient, with or without caregivers, established functional goals collaboratively. Based on patient-stated goals, SMART (Specific, Measurable, Achievable, Realistic, Timed) goal statements were agreed that reflected the anticipated level of achievement over an individualised timeframe [30]. The SMART framework offers a structured approach to formulate well-defined goals that can subsequently be reviewed to evaluate goal attainment [30].

The attainment of each goal was evaluated through a 6-point verbal rating scale that was converted into numeric ratings (-2 to + 2) for calculation [29]. A score of ‘0’ achievement of the goal as expected, while other levels include deterioration (-2) or partial (-1), somewhat better (+ 1) or much better (+ 2) achievement than expected. A GAS T-score 29 was computed for each participant using a standard formula to convert the ordinal data from the simple goal attainment scale ( -2 to + 2) to interval data for parametric statistical analysis. This provides a single evaluation of overall goal attainment for each person’s goals [27]. If all goals were achieved as expected the GAS T-score would be 50, but this is unlikely to occur in complex situations. Therefore, if goals are set in an unbiased fashion and allowing for equal over- and under-achievement of goals, the mean GAS T score would be 50 with a standard deviation (SD) of 10. A change in the mean GAS T-score of 10 is suggested to reflect a clinically significant improvement [29].

All study staff involved in setting goals with participants were trained in the goal setting process prior to data collection, in order to improve the quality and reproducibility of their goal setting practice [29]. This included allied health professionals (physiotherapists and occupational therapists), therapy assistants and nurses, from each study site. A consultant grade clinical-academic physiotherapist (SA) with extensive experience in goal setting (20 years, regular practice) led a full-day teaching program that covering goal negotiation, setting and reflection. Throughout the study, ongoing ad hoc supervision was offered via telephone/online calls and bimonthly feedback using quality ratings against agreed goal criteria was provided [29].

#### EuroQoL 5 dimension 5 levels (EQ-5D-5 L)

The EQ-5D-5 L is a patient-reported instrument used to measure health-related quality of life [31]. The EQ-5D encompasses five dimensions: mobility, self-care, usual activities, pain/discomfort and anxiety/depression. Each dimension offers five response options (no, slight, moderate, severe, extreme problems/unable) for patients to indicate their health status on a given day. Patients select one response option per dimension, resulting in a 5-digit health profile. Subsequently, a value set is applied to convert the responses into an index score. This measure was included to provide a global heath outcome against which the responsiveness of goal attainment could be compared.

### Mapping of goals

Text from patient stated goals and SMART goal statements were considered together and mapped using the International Classification of Functioning, Disability and Health (ICF) [32]. The ICF provides an internationally accepted framework and standardized language and coding system [33]. Mapping was conducted independently by two authors (LF, SA), then discussed together with a third (MM). Classification was at the first level, e.g. d4. Mobility and second level, e.g. d450 Walking [34] and the 2017 ICF Browser [35] was used as a guide. An element of interpretation was required, as described by others [24], and ICF linking rules were used to guide decisions and reach consensus [34, 36]. Goals could map onto several categories, in which case all were documented but the meaningful concept most relevant to the patient stated goal was used in the summative analysis. The SMART goal statement timeframe was summarised in days.

### Statistical analysis

Patient demographics and clinical characteristics were summarised using descriptive statistics; means (SDs) or medians (ranges) for continuous variables, and count (percent) for categorical variables, with a 95% CI where appropriate. GAS T-scores were summarised by mean (SD). The mean change in GAS T-score and EQ-5D pre- to post-rehabilitation, along with a 95% confidence interval, was then examined using paired t-tests. Missing post-rehabilitation data was assumed not at random, as loss to follow-up was expected to relate to clinical deterioration or death, therefore no attempt to impute data was made.

Ordinal logistic regression with the proportional odds assumption was used to identify factors associated with goal achievement. Achievement of each goal was summarised as a binary outcome achieved (0 to + 2) or not achieved (-2 to -1) as the dependent variable. Exploratory patient factors (age, sex, primary diagnosis [cancer/non-cancer], living arrangement, mobility status, Phase of Illness, AKPS, co-morbidities), service factors (inpatient/outpatient, home visit, group programme, staff input (1/≥2 professions), and rehabilitation intervention factors (symptom management, mobility, task practice, exercise programme, equipment provision, socialisation and communication, total number of interventions received, staff rated goal difficultly (not, a little, moderately, or very), type of goal (impairment, activity or participation), and goal timeframe were considered. For each group of variables (patient, service, intervention) explanatory variables significantly associated with the outcome (*p* < 0.10) in bivariate analysis were included in a multivariable model to produce adjusted odds ratios. Analyses were conducted using STATA version 16. All tests were 2-sided with an α value of *p* < 0.05.

Our target sample size of 300 was based on the precision to which goal attainment in the study population could be estimated. Assuming 15% attrition the 95% CI for any observed proportion of goal achievement would not exceed ± 6% with a large sample normal approximation (nQuery Advisor^®^, US). This sample size would provide sufficient power to reliably examine up to 30 dependant variables [37] in our regression modelling.

## Results

### Characteristics of participants

364 patients were recruited, 54% were female, with a mean (SD) age of 67.9 (13.8) years (Table 1). The majority had a primary cancer diagnosis (*n* = 257, 70.6%) and a median of 2 co-morbidities. Most participants were in the stable Phase of Illness (*n* = 257, 70.6%), with a median AKPS of 60, and able to ambulate with or without an aid (*n* = 332, 91.2%). Approximately two-thirds of participants (*n* = 233, 64.0%) were outpatient or community patients, and around one-third were living alone (*n* = 113, 31.0%).

**Table 1:** Characteristics of participants and rehabilitation (*n* = 364)

	*N* (%)
Age, mean (SD)	67.9 (13.8)
Sex	
Female Male	197 (54.0) 167 (46.0)
Ethnicity	
South Asian or Asian British Black, Black British, Caribbean or African Mixed or multiple White British White other Other	9 (2.5) 22 (6.0) 10 (2.7) 283 (77.7) 32 (8.8) 8 (2.2)
Primary diagnosis	
Cancer Chronic respiratory disease Cardiovascular Neurological	257 (70.6) 71 (19.5) 14 (3.8) 15 (4.1)
No of Co-morbidities: median [IQR]	2 [1–2]
Charlson Indices Score: median [IQR]	6 [2–12]
Australia-modified Karnofsky Performance status: median [IQR]	60 [50–70]
Palliative Phase of Illness	
Stable Unstable Deteriorating	257 (70.6) 59 (16.2) 48 (13.2)
Mobility status	
Independently mobile Mobile with walking aid Wheelchair-bound Bedbound	148 (40.7) 184 (50.5) 17 (4.7) 13 (3.6)
Living arrangement	
Lives alone Not living alone Receiving a care package	113 (31.0) 249 (68.0) 77 (21.0)
**Rehebilitaiton factors**	
Service	
Inpatient Outpatient / community	131 (36.0) 233 (64.0)
Providers	
Physiotherapist Occupational Therapist Rehabilitation Assistant Speech and language therapist Dietitian Volunteer Other, e.g. nursing assistant, complementary therapist Total no. of staff involved: median [IQR]	330 (90.7) 132 (36.3) 153 (42) 19 (5.2) 29 (8.0) 58 (15.9) 36 (9.8) 2 [1–3]
Interventions	
Symptom management (anxiety, breathlessness, dysphagia, fatigue, lymphoedema, nutrition, pain) General exercise programme Task practice (activities in daily living (ADLs), transfers, stairs) Mobility practice Equipment provision and carer training Socialisation and communication Other, e.g. creative arts, psychological support Total no. of interventions: median [IQR]	310 (85.0) 262 (72.0) 225 (62.0) 224 (61.5) 136 (37.0) 49 (13.0) 14 (3.8) 3 [2–5]

### Functional goals and intervention

Participants set a median (range) of 2 (1–4) goals each, resulting in a total of 645 goals. The median (range) timeframe for these goals was 28 (1-196) days. The primary focus of the SMART goal statements according to the WHO-ICF was distributed as follows: 51% on the level of activity, 29% on impairments to body structure and function, and 20% on participation. The goal areas covered 13 of the possible 30 WHO-ICF first level domains, most frequently relating to mobility, general tasks and demands, mental functions, community, social and civic life, and self-care. A frequency ranking of domains, with example patient stated and SMART goals, is shown in Table 2.

Rehabilitation interventions directed towards the goal(s) were provided by a median (range) of 2 (1–3) professional groups per patient. The most common providers were physiotherapists (90.7%), rehabilitation assistants (42%), and occupational therapists (36.3%). Intervention delivery primarily occurred through outpatient groups (34.5%) and/or clinics (25.7%), or inpatient wards (34.7%). A smaller proportion of participants received interventions in outreach community settings (18.6%), e.g. town halls. Participants received a median (range) of 3 (2–5) rehabilitation interventions, with the most common being symptom management (85%), exercise programmes (72%), task practice (62%), and mobility practice (61.5%) (Table 1).

### Goal attainment and health-related quality of life outcomes

**Fig. 1:**
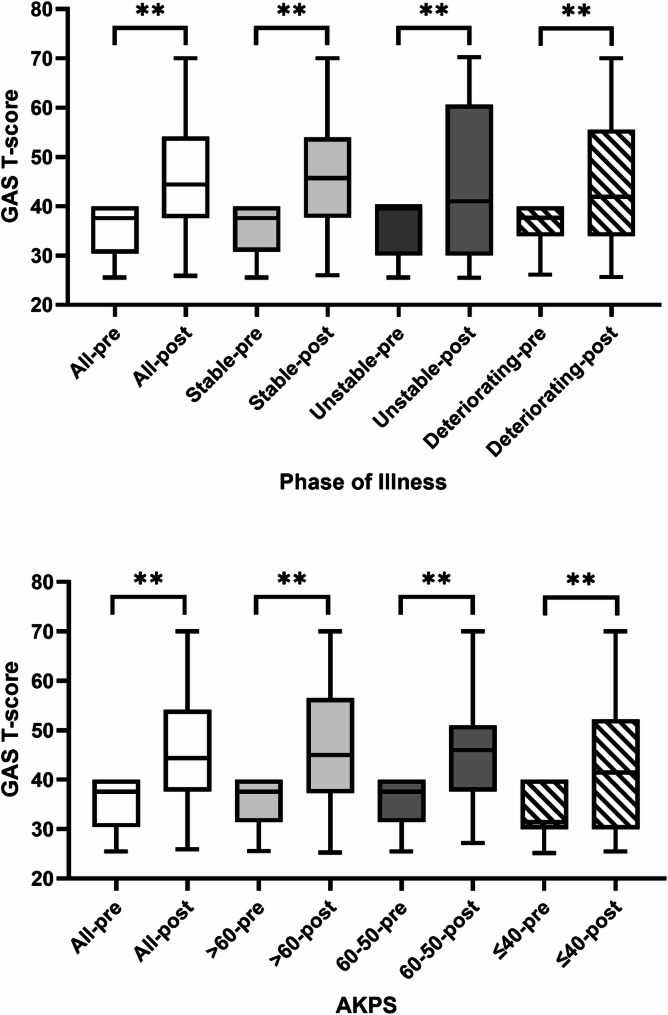
Goal attainment pre-to-post rehabilitation overall (*n* = 363) and according to palliative Phase of Illness and Australia-modified Karnofsky Performance Status at baseline. Boxplots show median [IQR] and whiskers show 95% CI, ** *p* < 0.01

**Fig. 2:**
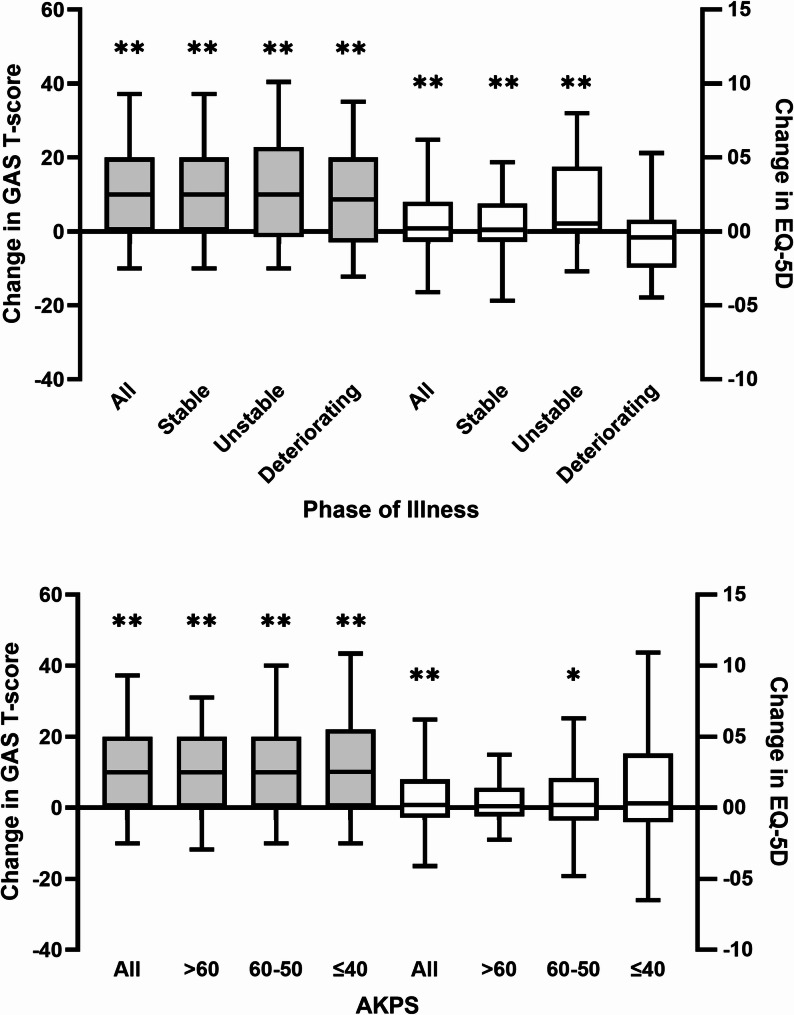
Change in GAS T-score and EQ-5D by palliative Phase of Illness and Australia-modified Karnofsky Performance Status (AKPS) at baseline (*n* = 363). Boxplots show median [IQR] and whiskers show 95% CI. * *p* < 0.05,** *p* < 0.01

A total of 343 participants completed a goal review, resulting in the evaluation of 614/645 goals (95.2%). The mean (SD) pre-and post-rehabilitation GAS T-scores were 35.6 (4.9) and 46 (14.4) respectively, with a mean (SD) change of 8.9 (13.4) (*p* < 0.01). There were statistically significant improvements in GAS T-scores overall and across all subgroups by Phase and AKPS following receiving rehabilitation interventions (*p* < 0.01) (Fig. 1). EQ-5D score improved overall (mean (SD) change 0.05 (0.27), *p* < 0.01) and for those with stable and unstable, but not deteriorating Phase of Illness. Scores improved in participants with AKPS 50–60 (*p* = 0.01) but not in other subgroups by functional status (Fig. 2).

### Factors associated with goal achievement

Overall, 310 (50.5%) goals were attained, while 304 (49.5%) were not. In univariable analyses (shown in supplementary material) patient factors related to goal attainment were living alone (OR = 1.80; 95% CI = 1.28, 2.54), being wheelchair or bedbound (OR = 0.42; 95% CI = 0.21, 0.82) and having three or more comorbidities (OR = 1.44; 95% CI = 0.94, 2.19). Associated service-related factors were inpatient status (OR = 1.80; 95% CI = 1.29, 2.54) and receiving an exercise intervention (OR = 0.57; 95% CI = 0.38, 0.84) or multiple interventions (OR = 1.19; 95% CI = 1.08, 1.30). Table 3 presents the results of the multivariable analysis. Adjusting for patient-, service-, and intervention-related factors brought forward, living alone (OR = 1.70, 95% CI = 1.18, 2.44), and receiving multiple interventions (OR = 1.19; 95% CI = 1.08, 1.30) were significantly associated with a higher likelihood of goal attainment. Conversely, being wheelchair or bedbound (OR = 0.32, 95% CI = 0.15, 0.71), receiving a general exercise intervention (OR = 0.57; 95% CI = 0.38, 0.84), having goals rated as of extreme difficulty (OR = 0.13; 95% CI = 0.03, 0.50) were significantly associated with a lower likelihood of goal attainment (Table 3).

**Table 2:** Goal mapping ranked by WHO-ICF domain

ICF domain (code)	Frequency *n* (%)	Example patient-stated goal	Example SMART goal statement
d4. Mobility (e.g. walking, getting around, transportation)	114 (18)	Walk to paper shop	To walk to paper shop independently with 4-wheeled walker 3x/week in 6 weeks
d2. General tasks and demands (e.g. stairs, transfers)	100 (16)	To return home with my dog	To be discharged home to microenvironment with her dog, with assistance of package of care and neighbour in 2 weeks
b1. Mental functions (e.g. anxiety, confidence, fatigue)	100 (16)	Fatigue - to reduce post bath/shower	Improve fatigue level after having a bath/shower from 0/10 by 2 points on Numerical Rating Scale in 6 weeks
d9. Community, social and civic life (e.g. hobbies, social activities, hospital appointments)	99 (15)	To go to the garden in wheelchair	To be able to transfer into a wheelchair using equipment and assistance as needed to enable a visit to the gardens in 2 weeks
d5. Self-care (e.g. washing, dressing, toileting, eating, drinking)	68 (11)	To be able to transfer independently to the toilet using aid as necessary	Be able to transfer independently via standing, to the toilet, using aids as necessary. − 6 weeks
d6. Domestic life (shopping, housework, cooking)	46 (7)	To be more independent and shop for herself	In 8 sessions be able to walk with aid to local shop twice in same week to do her own food shopping
b4. Functions of the cardiovascular, haematological, immunological and respiratory systems (e.g. breathlessness)	33 (5)	To climb stairs without losing breath	Reduce my severity of breathlessness from 10/10 to 8/10 Borg after climbing stairs in 5 weeks
b2. Sensory functions and pain (e.g. balance, pain control)	26 (4)	Less pain when walking around	To decrease pain on walking according to Visual Analogue Scale of 7/10 by 3 points in 8 weeks
d7. Interpersonal interactions and relationships (e.g. with family, friends, health professionals)	25 (4)	Take the children to school	Take both children to school 3 times a week in 4 weeks
b7. Neuromusculoskeletal and movement related functions (e.g. muscle strength, flexibility, tremors)	24 (4)	Improve strength in legs	To increase muscle power in hip flexors and knee extensor to 3/5 and 5/5 (Oxford Scale) in 4 weeks.

**Table 3:** Multivariable regression analysis of factors associated with goal achievement following personalised rehabilitation (*n* = 614)

				OR (95% CI)
**Facilitators**				
Lives alone				1.70 (1.18–2.44)*
Service summary (Inpatient, outpatient used as reference category)				1.48 (0.97–2.26)
Total number of interventions				1.19 (1.08–1.30)*
**Barriers**				
Baseline mobility (independently mobile used as reference category)		Mobile with walking aid		0.80 (0.55–1.16)
		Wheelchair/bed bound		0.32 (0.15–0.71)*
**General exercise programme**				0.57 (0.38–0.84)*
Difficulty (‘not’ used as reference category)		A little		0.49 (0.15–1.67)
		Moderately		0.31 (0.09–1.02)
		Very		0.13 (0.03–0.50)*

*p < 0.05

## Discussion

This study details the characteristics of functional goals among people receiving palliative care. Our findings reveal that most goals in this context focus on activity and participation, within a short timeframe of one month or less. Progress towards personalised goals was consistently achieved following rehabilitation, across the predominant Phases of Illness and functional status groups, including those who were deteriorating or largely confined to bed.

Functional goals spanned 13 of the possible first level 30 WHO-ICF domains, most frequently relating to mobility, general tasks and demands, mental functions, community, social and civic life, and self-care. A large majority (71%) concerned activity and participation, which is consistent with previous research examining therapeutic goals for patients undergoing acute or chronic rehabilitation [38–42]. This reflects the priorities among individuals with serious illness, which extend beyond the body and encompass meaningful activity, community engagement and social integration [5, 7]. Specifically, 20% of the goals involved participation in a life situation, which is substantially higher than 6.6% in chronic rehabilitation but lower than the 24.6–31.7% reported in acute rehabilitation services [43, 44]. These differences may be attributed to differing characteristics of the populations, including the severity of impairment, stage of recovery and prognosis, as well as the available rehabilitation.

Contrary to the findings of a recent scoping review on goal-setting in rehabilitation, which identified a tendency for patients to establish long-term goals [45], our study found that over half of the goals set within palliative rehabilitation services had a timeframe of one month or less. This discrepancy could be attributed to the differences in patient expectations and prognosis. Our population tended to set goals to address their immediate needs and facilitate a sense of accomplishment or successful adaptation, rather than focusing on the longer-term [21, 46]. This emphasis on short-term goals could be a particular feature of the rehabilitation experience in palliative care. Notably, we found consistent levels of goal attainment by participants across all included Phases of Illness and functional status. This included patients who were deteriorating and/or confined to bed, whose goals were often set for the next days. Though the mean change in GAS T-score of 8.9 was marginally below the clinically significant threshold of 10 [29], this could reflect the overall declining trajectory of our study population. We did observe concurrent improvements in quality of life for most individuals, which suggests perceived benefit and successful alignment of rehabilitation interventions to goals.

Several patient and intervention-related factors were independently associated with goal achievement. Patients who were living independently demonstrated a higher likelihood of achieving their goals. While family involvement can streamline the processes of goal setting, family members introduce their personal agendas that may not align with the patient’s. This can disrupt and impede goal attainment [47]. Individuals living alone may also possess heightened motivation to retain their independence, thereby increasing engagement with rehabilitation towards the goals they set. Furthermore, patients were less likely to achieve goals when they joined general exercise programs, e.g. group circuits, as rehabilitation interventions. This may be attributed to the lack of specificity of the intervention relative to others, e.g. task practice or provision of an assistive device, or a tendency to align goal timeframes to the programme rather the individual’s situation.

### Implications for clinical practice

In palliative care, patients encounter varied and unpredictable challenges as disease progresses. Goal setting in this context necessitates a balanced approach to agree on goals that are meaningful yet achievable within the constraints of poor or uncertain prognosis. Goal Attainment Scaling (GAS) offers a structured approach to identify what matters most to each patient and then direct interventions towards those aspects of functioning [28]. The 6-point rating scale used to evaluate attainment may be particularly suited to this population, as it recognizes partial achievement of a goal that would not be acknowledged if goals were assessed in a binary manner (i.e. achieved or not). GAS is a system of evaluating achievement of the goals of intervention set by the patient and clinical team before the starting treatment. The method therefore provides both a quantification of individual goal outcome and qualitative information about the specific goals set. It is therefore an evaluation of expected goal achievement, dependent on the patient’s ability to change and the clinical team’s ability to predict that change [29].

Limitations have been identified in using the standard GAS approach which relate to comparison of scores between individuals or groups and data obtained being ordinal rather than interval, undermining the validity of the calculation of the T score [48]. Without the application of SMART goal statements however, the systematic evaluation of goals becomes highly subjective and it is therefore critical to this approach. Criticism has been directed at GAS because of the constraint this structured approach places on the goal setting process [14, 15], though as demonstrated in a number of studies using GAS, dynamic and challenging goals are still set in rehabilitation with positive treatment outcomes [49–52]. By personalising rehabilitation providers can increase the likelihood of goal attainment, though this can also be affected by external factors, e.g. changes in local weather or systems they work within, e.g. late delivery of assistive products. Nonetheless, the goal setting process itself can be valuable, irrespective of the method used, as working towards goals can contribute to well-being in palliative care [21].

### Methodological reflection

This study included a diverse patient population with advanced or progressive conditions accessing real-world rehabilitation services in palliative care. With structured training and supervision rehabilitation providers were able to set functional goals with consecutive patients, across different settings. The vast majority of goals could be reviewed (94.2%), including for people in the last days of life, where goal attainment could be established using clinical note review. The overall level of and variation in goal achievement highlights the high degree of uncertainty in this context and the complexities in predicting rehabilitation outcome.

Our mapping of goals onto the ICF involved a degree of subjectivity and interpretation. We used both the patient stated and SMART goal for this purpose, as there is a risk that meaningful concepts stated by the patient are obscured as clinicians bring in aspects of measurement. Use of multiple reviewers, who undertook ICF e-learning and were guided by linking rules, aided the standardisation of this process. Nonetheless, there were instances where discussion was required to reach consensus and there may have been a bias to align with the more experienced reviewer (SA). Others have used the ICF as a tool in the goal setting process itself [53]. This would prevent the need to map externally but would require all clinicians to be familiar with the ICF and may restrict the patient-clinician interaction by introducing technical and complex language.

Finally, we categorized rehabilitation interventions using a framework developed with staff across sites. The comparability of our findings could have been enhanced if interventions were categorised using recognized frameworks, for example the glossaries of interventions within the WHO Package of Interventions [54]. Most of our categories translate across but the omission of education, advice and support for self-management is acknowledged, an aspect frequently emphasized in palliative rehabilitation contexts. Unpacking of complex interventions within ‘symptom management’ would have improved this aspect of our work.

## Conclusion

This study determined characteristics of functional goals in palliative care and factors associated with achieving them across a diverse hospice patient population. Functional goals in this setting typically focus on optimising activity and participation in the short term. Progress towards personalised goals can be achieved through personalised rehabilitation, including among people with deteriorating health or largely confined to bed. Our findings highlight the value of a person-centred approach to goal setting, in order to direct rehabilitation to address patients’ immediate needs and priorities impacting their quality of life. Goal Attainment Scaling offers a practical means to direct and evaluate the rehabilitation process in palliative care, as well as support individualised outcome assessment through incorporation of measures in the goal setting process. Overall, this study highlights the value of multi-professional palliative care that includes rehabilitation as an integral element to optimise functioning, well-being and quality of life.

## Electronic supplementary material

Below is the link to the electronic supplementary material.

Supplementary material 1

## Data Availability

The datasets used and/or analysed during the current study are available from the corresponding author on reasonable request.
