# Supplementary material for: Functional goals and outcomes of rehabilitation within palliative care: a multicentre prospective cohort study
Source: BMC Palliat Care. 2025 Jul 1;24:172. doi: 10.1186/s12904-025-01816-0 (PMC12210815; doi:10.1186/s12904-025-01816-0)
Supplement: Supplementary file 1 — Supplementary material 1 [file 12904_2025_1816_MOESM1_ESM.docx]

**Supplementary Material**

The following tables detail the ordinal logistic regression used to identify factors associated with goal achievement. For each group of exploratory variables (patient, service, intervention) bivariate analysis is shown in Model 1, then explanatory variables associated with goal achievement were combined in a multivariable Model 2. Following adjustments for other patient/service/intervention, these factors were then included in a final multivariable model (shown here and in the main text).

**Patient factors**

Binary outcome variable (0 = goal not achieved, 1 = goal achieved) regressed onto explanatory variable(s) in logistic regression models

| (N=614) | | OR (95% CI) | |
| --- | --- | --- | --- |
|  | | **Model 1** | **Model 2** |
| Age (mean) |  | 0.99 (0.98-1.01) | -- |
|  |  |  |  |
| Sex (0 = male, 1 = female) |  | 1.15 (0.84-1.58) | -- |
|  |  |  |  |
| Diagnosis (cancer used as reference category) | Cardiovascular | 1.20 (0.51-2.84) | -- |
|  | Respiratory | 1.09 (0.73-1.65) | -- |
|  | Neurodegenerative | 0.67 (0.29-1.52) | -- |
|  | Other | 1.80 (0.59-5.46) | -- |
|  |  |  |  |
| Lives alone |  | 1.80 (1.28-2.54)** | 1.72 (1.21-2.43)** |
|  |  |  |  |
| Baseline mobility (independently mobile used as reference category) | Mobile with walking aid | 1.07 (0.77-1.50) | 1.01 (0.72-1.42) |
|  | Wheelchair/bed bound | 0.42 (0.21-0.82)** | 0.42 (0.21-0.83)** |
|  |  |  |  |
| Pre-rehab POI (‘Stable’ used as reference category) | Unstable | 0.83 (0.52-1.34) | -- |
|  | Deteriorating | 0.90 (0.57-1.43) | -- |
|  |  |  |  |
| Pre-rehab KPS (‘0-50’ used as reference category) | 60-70 | 1.23 (0.86-1.76) | -- |
|  | 80-100 | 0.91 (0.56-1.49) | -- |
|  | |  |  |
| Multimorbidity’s (3+ comorbidities compared to <3 comorbidities) |  | 1.44 (0.94-2.19)* | 1.33 (0.86-2.04) |
|  | |  |  |
| Charlson score (Mean) | | 0.99 (0.95-1.02) | -- |

*p<0.10, **p<0.05

**Service factors**

| (N=614) | | | | OR (95% CI)  Model 1 | | |
| --- | --- | --- | --- | --- | --- | --- |
| Home visits (service) | |  | | | 0.88 (0.58-1.32) | |
|  | | | | | | |
| Group programme (service) | |  | | | 0.79 (0.57-1.08) | |
|  |  |  |  | | |  |
| Service summary (In/Out) | |  | | | 1.81 (1.29-2.54)** | |
|  |  |  |  | | |  |
| No. of AHPs involved (‘0’ used as reference category) | | 1 | | | 2.94 (0.77-11.16) | |
|  |  | 2 | | | 2.78 (0.73-10.60) | |
|  |  | 3+ | | | 3.69 (0.97-14.01) | |

*p<0.10, **p<0.05

(As only one service factor was associated with the outcome bivariately, there was no need for an adjusted service factor model)

**Intervention factors**

| (N=614) | | OR (95% CI) | | | | |
| --- | --- | --- | --- | --- | --- | --- |
|  |  |  | | **Model 1** | | **Model 2** |
| Symptom management |  |  | | 1.09 (0.79-1.50) | | -- |
|  | | | | | |  |
| Mobility |  |  | | 1.54 (1.10-2.15)** | | 1.24 (0.79-1.96) |
|  |  |  |  | |  |  |
| Task practice |  |  | | 1.67 (1.22-2.30)** | | 1.02 (0.65-1.59) |
|  |  |  |  | |  |  |
| Exercise intervention |  |  | | 0.75 (0.54-1.05)* | | 0.67 (0.45-1.00)* |
|  |  |  |  | |  |  |
| Positioning, equipment and training | |  | | 1.79 (1.28-2.52)** | | 1.17 (0.74-1.86) |
|  |  |  |  | |  |  |
| Socialisation and communication | |  | | 1.49 (0.95-2.34)* | | 1.14 (0.66-1.97) |
|  |  |  |  | |  |  |
| Total number of interventions | |  | | 1.15 (1.06-1.24)** | | 1.14 (0.98-1.32)* |
|  |  |  |  | |  |  |
| Functional level |  |  | | 1.11 (0.78-1.60) | | -- |
|  |  |  |  | |  |  |
| Difficulty (‘Not’ used as reference category) | | A little | | 0.44 (0.14-1.42) | | 0.45 (0.13-1.51) |
|  |  | Moderately | | 0.30 (0.10-1.94)** | | 0.29 (0.09-0.96)** |
|  |  | Very | | 0.14 (0.04-0.47)** | | 0.10 (0.03-0.38)** |
|  |  |  |  | |  |  |
| Number of days |  |  | | 0.99 (0.98-1.00)** | | 0.99 (0.99-1.00) |
|  |  |  |  | |  |  |
| Type of goal (‘A’ used as reference category) | | I | | 1.02 (0.70-1.49) | | -- |
|  |  | P | | 0.92 (0.61-1.38) | | -- |

*p<0.10, **p<0.05

## Multiply adjusted model including all variables associated with outcome in multivariable models (p<0.1)

| (N=614) | | | | OR (95% CI) | |
| --- | --- | --- | --- | --- | --- |
| Lives alone | |  | | 1.70 (1.18-2.44)** | |
|  | | | | | |
| Baseline mobility (independently mobile used as reference category) | | Mobile with walking aid | | 0.80 (0.55-1.16) | |
|  |  | Wheelchair/bed bound | | 0.32 (0.15-0.71)** | |
|  |  |  |  | |  |
| Service summary (In/Out) | |  | | 1.48 (0.97-2.26)* | |
|  |  |  |  | |  |
| Exercise intervention |  |  |  | 0.57 (0.38-0.84)** | |
|  | |  | |  | |
| Total number of interventions | |  | | 1.19 (1.08-1.30)** | |
|  | |  | |  | |
| Difficulty (‘Not’ used as reference category) | | A little | | 0.49 (0.15-1.67) | |
|  |  | Moderately | | 0.31 (0.09-1.02)* | |
|  |  | Very | | 0.13 (0.03-0.50)** | |

*p<0.10, **p<0.05
